# Axillary Approach for Venoarterial Extracorporeal Membrane Oxygenation Cannulation

**DOI:** 10.7759/cureus.7788

**Published:** 2020-04-22

**Authors:** Talha Ahmed, Ayesha Safdar, Diljon Chahal

**Affiliations:** 1 Internal Medicine, University of Maryland Medical Center, Baltimore, USA; 2 Internal Medicine, Army Medical College, Rawalpindi, PAK; 3 Cardiology/Internal Medicine, University of Maryland, Baltimore, USA

**Keywords:** infective endocarditis, valve surgery, valve replacement surgery, extracorporeal membrane oxygenation support, cannulation, mechanical circulatory support

## Abstract

Extracorporeal membrane oxygenation support (ECMO) is a form of mechanical circulatory support that is used in patients with severe dysfunction of heart or lung or both. Depending on whether it is venovenous or venoarterial support, it can temporarily substitute for circulation and ventilation while the underlying cause is addressed. Traditional approach for cannulation usually involves the femoral vessels. This is due to the easy accessibility, larger lumen of vessels, and physician expertise and training in femoral approach. However, in certain circumstances like critical lower extremity ischemia, crush injury or trauma to lower extremity, and lower extremity infections (like necrotizing fasciitis), this approach is not practical. In these situations, axillary vasculature provides a good substitute for ECMO cannulation.

## Introduction

In states of severe circulatory collapse, some form of temporary support is required to sustain life while the underlying cause is being addressed. This essentially is the role of mechanical circulatory support, of which one kind is extracorporeal membrane oxygenation support (ECMO) [1]. Using ECMO we can bypass the lungs (in venovenous or VV-ECMO) or both the lungs and the heart (in venoarterial or VA-ECMO) support [2,3]. Our patient had a combined cardiogenic shock and septic shock leading to combined heart/lung failure. We utilized VA-ECMO support to sustain life in our patient while the underlying cause was being addressed. A unique situation in our case was presence of severe lower limb ischemia from a global state of low perfusion and also iatrogenic from prolonged femoral sheath placement for percutaneous coronary intervention. To overcome this problem, axillary artery and femoral vein were utilized for obtaining access for VA-ECMO.

## Case presentation

**Figure 1:**
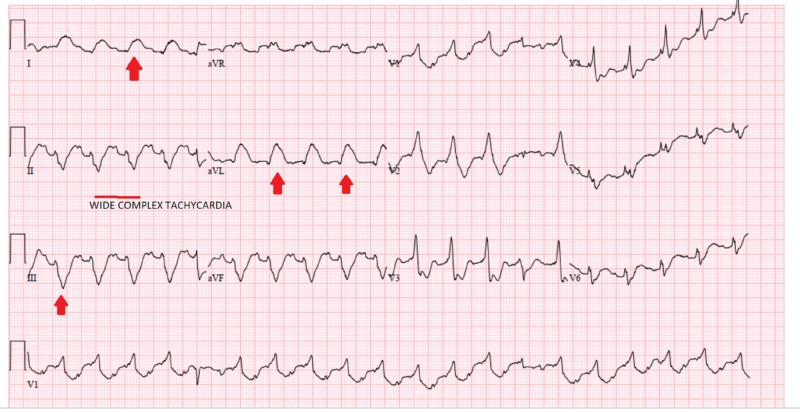
Electrocardiogram with persistent ventricular arrhythmias despite restoration of blood flow after cardiac catheterization

A 31-year-old female with a past history of endocarditis status post mitral prosthetic valve repair an year ago and continued intravenous drug use presented to the emergency department with altered mental status. On initial arrival, the patient was found to be hypotensive with systolic blood pressures of 50 mmHg and 60 mmHg, a heart rate of 110 beats per minute, a temperature of 38.1 degree Celsius with mild respiratory distress, and an oxygen saturation of 95% on room air. There was difficulty in establishing vascular access due to severe vasoconstriction (increased peripheral vascular resistance from shock); however, ultrasound-guided vascular access was ultimately established. The 12-lead electrocardiogram showed ST-segment elevations in high lateral leads, and cardiac catheterization lab was activated immediately after loading aspirin, clopidogrel, and statin therapeutic heparin drip. On left heart catheterization (LHC), she was found to have an embolus lodged in her left main coronary artery which was removed. When flow was restored, an intra-aortic balloon pump (IABP) was placed due to severe refractory hypotension despite inotrope and vasopressor support with dobutamine and norepinephrine, respectively. However, the patient continued to experience ventricular arrhythmias on the monitor and on electrocardiogram (Figure 1).

**Figure 2:**
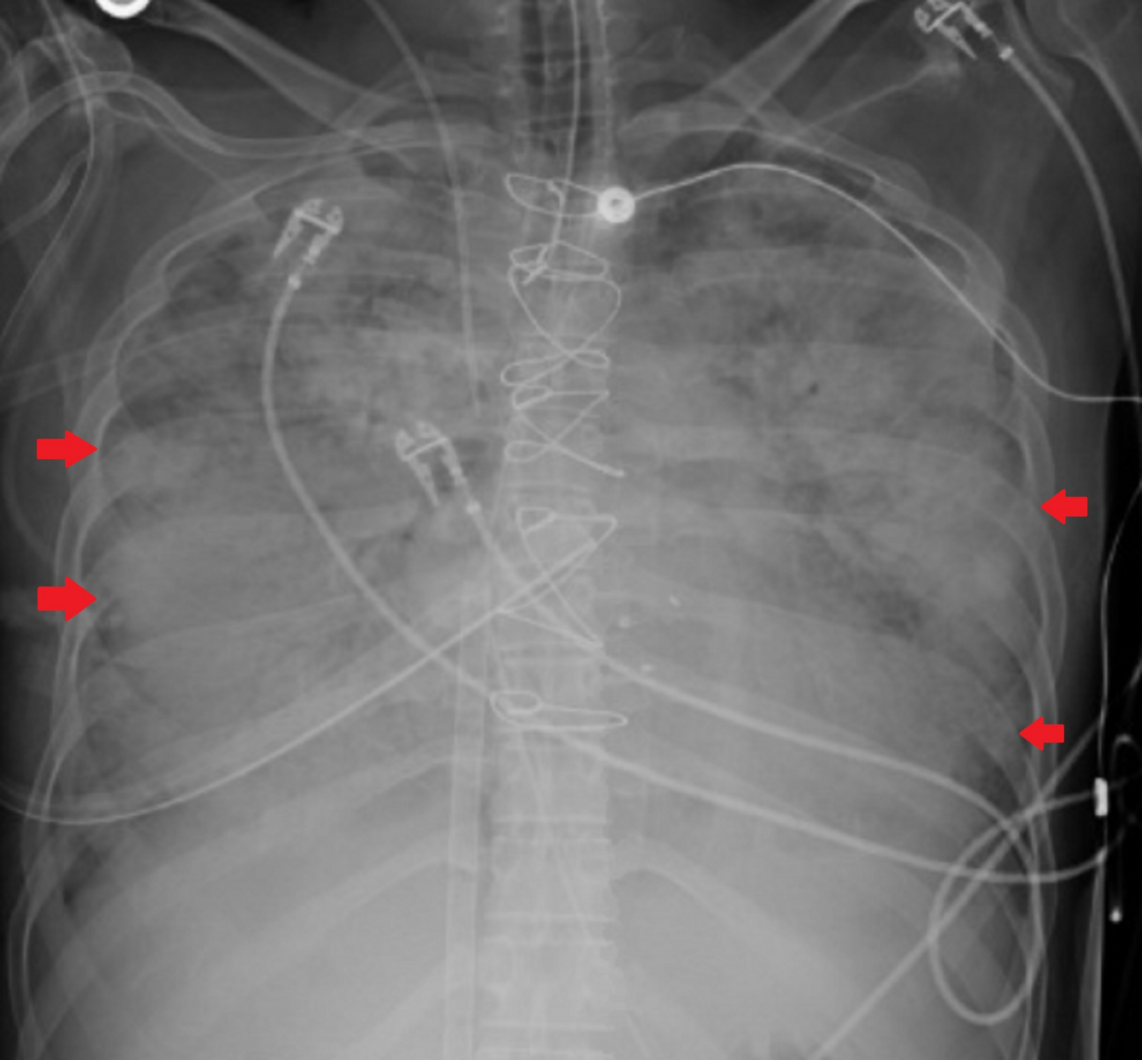
Chest x-ray with worsening bilateral pulmonary infiltrates

A tentative diagnosis of severe mixed shock (cardiogenic and septic) was made with the source suspected to be a probable prosthetic valve endocarditis (PVE) due to continued drug use. The patient was started on broad-spectrum antibiotics in addition to hemodynamic support. Due to worsening hypoxia and respiratory distress, she was intubated and mechanically ventilated. Her chest x-ray showed worsening bilateral pulmonary infiltrates (Figure 2).

**Figure 3:**
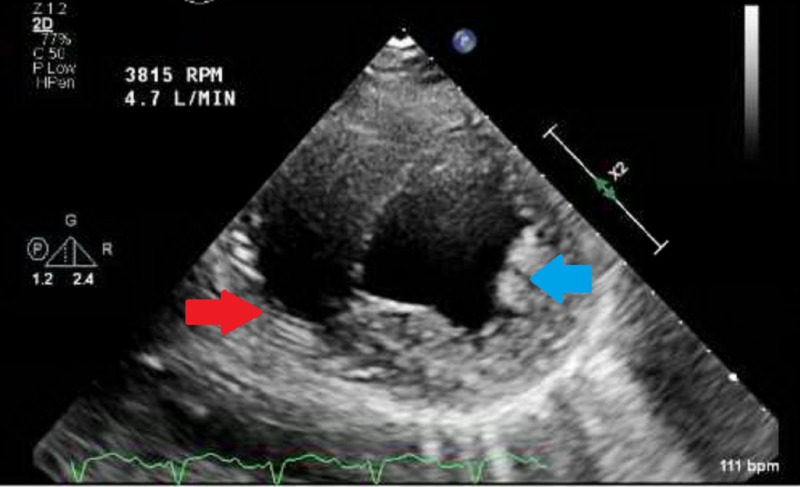
Transthoracic echocardiogram with severely dilated right ventricle (red arrow) and left ventricle (blue arrow) in short axis view

A transthoracic echocardiogram revealed severely depressed right and left ventricular function with mitral and aortic stenosis but with no obvious vegetations on the valves including the prosthetic mitral valve (Figure 3).

**Figure 4:**
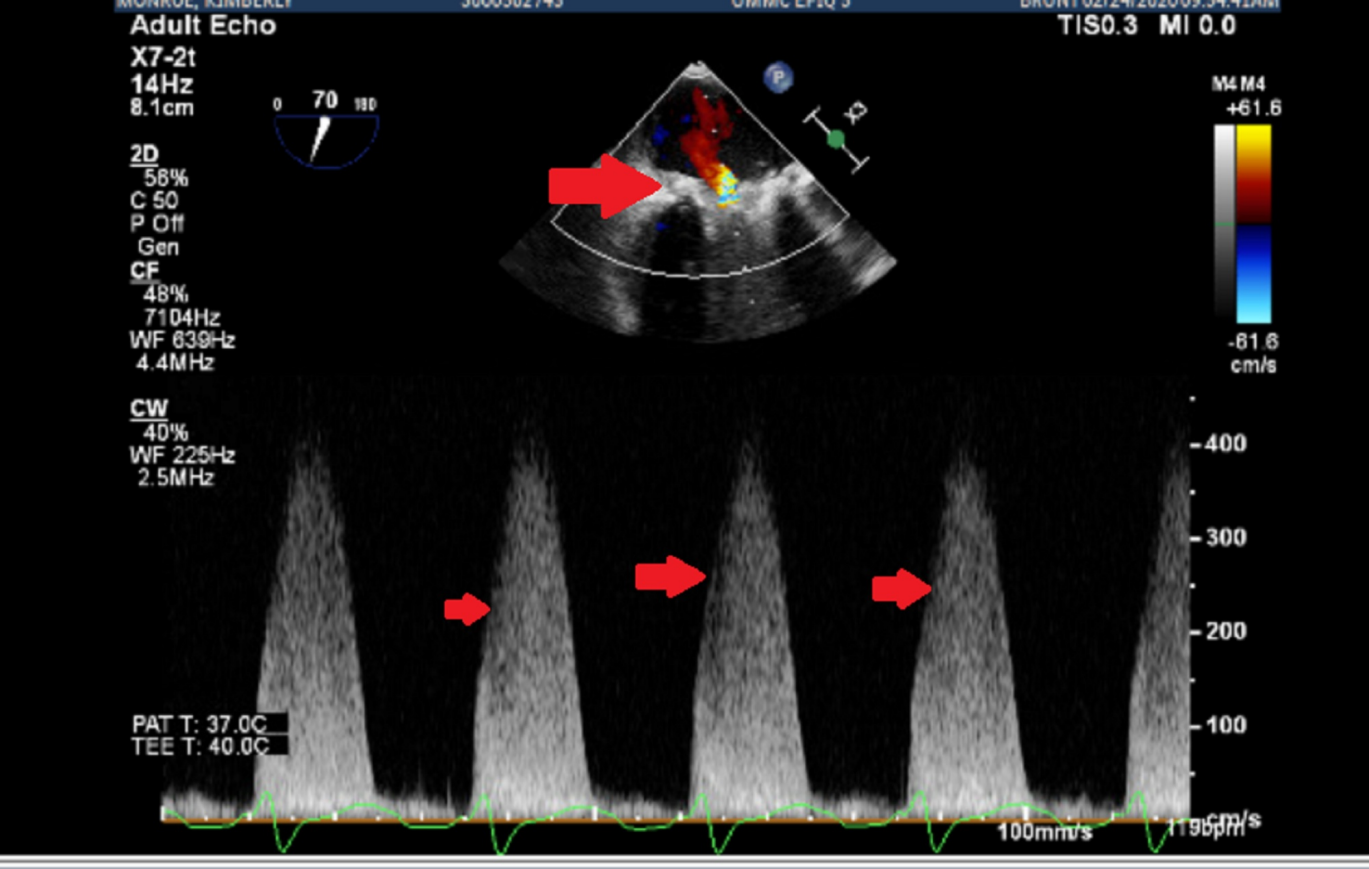
Transesophageal echocardiogram with severe mitral stenosis with elevated gradients across the prosthetic mitral valve

**Figure 5:**
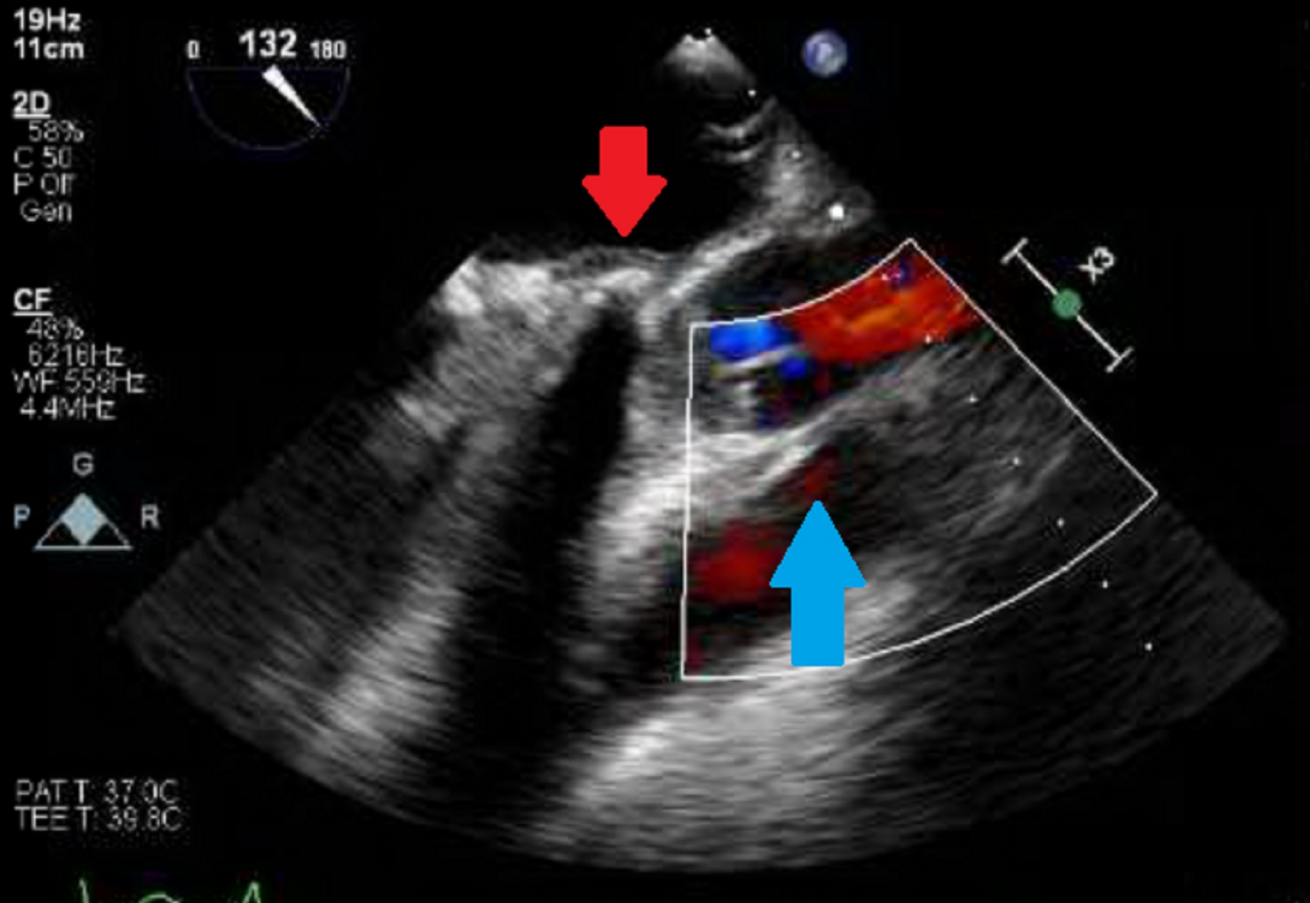
Transesophageal echocardiogram with severe stenosis and vegetation on prosthetic mitral valve (red arrow) and severe aortic stenosis (blue arrow)

However, despite all these efforts her clinical condition continued to deteriorate with worsening hypotension and hypoxia. Due to persistent hypotension and bilateral pulmonary infiltrate, it was decided to cannulate her for VA-ECMO. Femoral vessels were very difficult to access due to limb ischemia from a profound state of increased peripheral vascular resistance. This was compounded by the fact that a vascular sheath was kept in her left femoral artery for 14 hours to monitor the arterial pressure during the LHC procedure and at the time of IABP placement and afterwards in the critical care unit. This made the suspicion of compartment syndrome of the left leg a possibility and close monitoring was required. Hence, as an alternative, the patient was successfully cannulated using right axillary artery for outflow cannula (15 French) and right femoral vein for inflow cannula (25 French). The IABP was removed during the same procedure. Due to worsening pulmonary edema, an atrial septostomy was performed for left ventricular shunting on the next day. A transesophageal echocardiogram was done which revealed a mobile vegetation on the mitral valve with severe mitral and aortic stenosis (Figures 4, 5).

However, due to a high surgical risk and lack of improvement in clinical status despite advanced hemodynamic support, comfort care was deemed appropriate after discussion with the family. Ultimately, life support was terminated at the request of the family and the patient died. 

## Discussion

There has been a recent increase in utilization of ECMO during the last decade in major centers all across the world including Unites States. The basic principle is to offload or bypass the heart and the lungs depending on which organ is affected. In lung disease with a normal functioning heart, a VV-ECMO takes venous blood from one of the major veins, which is then circulated through the machine where gas exchange takes place and the oxygenated blood is returned to another vein from where it returns to the heart to be pumped to the whole body [4,5]. In both heart and lung disease, as in our patient, a VA-ECMO will take blood from one of the major veins which will then pass through a machine where not only gas exchange will happen but it will also pump the oxygenated blood back to one of the major arteries to be distributed to the whole body. Femoral vessels are most commonly utilized for ECMO as they are easily approachable, have large lumen to accommodate the ECMO cannulas, and can be accessed percutaneously and require less expertise. However, they are also prone to complications including lower limb ischemia from prolonged ECMO cannulation [6,7].

Using VA-ECMO the increased blood volume returning to the arterial system usually increases the afterload on the already compromised heart. This can be mitigated by shunting blood from left ventricle using impella, balloon pump, or atrial septostomy [8].

Percutaneous cannulation of the femoral vessel allows for rapid initiation of support; however, the femoral arterial cannulae can be partially or totally occlusive, and perhaps lead to distal limb ischemia. This is particularly true for patients who already have reduced limb perfusion in setting of shock. To limit vascular limb complications, alternative cannulation sites have been considered. Few reports are available describing the role of the subclavian or axillary artery in the past [9]. Axillary artery cannulation is a viable alternative method for preserving cardiac blood flow and limiting the risks of femoral artery cannulation. 

One consideration however is that axillary artery access is not practical in emergency circumstances because they require open surgery. It can be used in lung or heart transplant procedures which are planned. However, in emergent situations it is not practical. In our patient, axillary artery cannulation provided a safe and effective method for V/A-ECMO support. The inflow site was femoral vein and the outflow site was axillary artery. However, the patient ended up having PVE, and due to high surgical risks surgery could not be performed for valve replacement. There are numerous case reports on multiple percutaneous valve repair therapies under ECMO support [10]. However, in PVE, the valve needs surgical replacement and cannot be repaired percutaneously, hence rendering it a high-risk procedure. There also have been previous studies reporting the use of subclavian approach for ECMO cannulation [11]. However, in our case, despite successful cannulation using the axillary artery approach, patient's comorbidities and high risk made surgery prohibitive.

## Conclusions

PVE is a condition with high morbidity and mortality. In patients with refractory shock, ECMO can serve as a bridge while the infection is treated. However, shock is a state of low tissue perfusion that sometimes makes lower extremity cannulation impractical particularly in setting of any iatrogenic cause of lower limb ischemia. Axillary artery cannulation provides a safe and effective method for VA-ECMO support in such scenarios. However, it is more feasible for planned surgeries and less desirable for emergent cases as it requires surgical approach and is not accessible percutaneously. In certain cases of lower extremity ischemia and concerns for compartment syndrome, axillary artery cannulation can be performed on an urgent basis. 
